# Portuguese Workers of Private Institutions of Social Solidarity and Affective Job Satisfaction: An Exploratory and Confirmatory Factor Analysis

**DOI:** 10.3390/ejihpe15100192

**Published:** 2025-09-24

**Authors:** Silvia Silva, Ricardo Pocinho, Maria José Rodriguez Conde, Gabriela Topa, Juan José Fernández Muñoz

**Affiliations:** 1 Polytechnic Institute of Leiria, Centro Interdisciplinar em Ciências Sociais CICS NOVA, 2400 Leiria, Portugal; silvia.c.silva@ipleiria.pt (S.S.); ricardo.pocinho@ipleiria.pt (R.P.); 2 University Institute of Educational Sciences, Salamanca University, 37001 Salamanca, Spain; mjrconde@usal.es; 3 Department of Social and Organizational Psychology, Universidad Nacional de Educación a Distancia (UNED), 28040 Madrid, Spain; gtopa@psi.uned.es; 4 Area of Methodology of Behavioral Sciences, Universidad Rey Juan Carlos, 28922 Alcorcón, Spain

**Keywords:** affective satisfaction, validation, emotional exhaustion, factor analysis, depersonalization

## Abstract

This study evaluates the validity and factorial structure of the affective job satisfaction scale (BIASJ) among 234 workers from private institutions of social solidarity (IPSS) in Portugal. Emotional job satisfaction, a key marker of psychological well-being, is associated with positive outcomes for employees and organizations. The sample was mainly female, with an average age of 39.15 years (SD = 8.22). The BIASJ and Maslach burnout inventory (MBI) measured job satisfaction and burnout. The BIASJ demonstrated high internal consistency (Cronbach’s alpha = 0.87, McDonald’s omega = 0.88) and a unidimensional structure. Significant negative correlations with emotional exhaustion and depersonalization supported its criterion validity. The results confirm the BIASJ as a reliable instrument for assessing job satisfaction in IPSS settings in Portugal. Future research should incorporate more diverse, gender-balanced samples and utilize probability sampling to improve generalizability.

## 1. Introduction

Emotional job satisfaction is regarded as a key indicator of psychological well-being within organizations and has been linked to numerous positive outcomes for both employees and companies (Brayfield & Rothe, 1951). This concept describes the personal experience of enjoyment and contentment at work and is connected to the emotions and positive feelings we encounter in the professional environment and during our interactions (Lodi et al., 2021; Warr, 2007; Topa & Alcover, 2015).

This concept differs from cognitive satisfaction, which is based on more rational evaluations of objective work factors like salary or job security. Emotional satisfaction, on the other hand, is linked to the emotional responses that arise from daily professional activities. The literature shows that workers who experience high levels of emotional satisfaction tend to demonstrate greater motivation, job commitment, and resilience when facing challenges (Judge & Kammeyer-Mueller, 2012). Additionally, emotional satisfaction is directly connected to productivity, job performance, and the intent to stay in the organization, which reduces turnover and absenteeism (Platania et al., 2021; Spector, 1997). Conversely, a work environment filled with stressors such as excessive workload, lack of recognition, and difficulties in interactions with colleagues and supervisors can negatively affect emotional satisfaction and increase employees’ vulnerability to burnout. Several authors have defined burnout as a state of emotional exhaustion, depersonalization, and diminished professional achievement that results from chronic work stress (Freudenberger, 1974; Maslach et al., 1996; Maslach & Leiter, 2016). Studies indicate that low emotional satisfaction is a significant predictor of burnout, underscoring the importance of understanding and monitoring this variable in the organizational setting (Romano et al., 2021).

For employees of private institutions of social solidarity (IPSS), emotional satisfaction plays an even more vital role, as these professionals often face high emotional demands due to direct interactions with vulnerable populations. The emotional toll of their work, along with challenges like limited resources and potential workload overload, can impact their well-being and influence the quality of services they provide (Platania et al., 2024). In the context of Portuguese IPSS, the workforce is highly diverse, including social workers, social educators, psychologists, nurses, direct care staff in residential facilities for the elderly (ERPI), home support assistants, and educators in day and social centers. These professionals work with vulnerable groups and face emotionally demanding situations that require both technical expertise and emotional resilience. This diversity highlights the complexity of the IPSS environment and emphasizes the importance of assessing affective job satisfaction among its staff. The evidence from Portuguese IPSS settings shows that higher job satisfaction is linked to less presenteeism and lower psychological distress among employees (Maurício & Laranjeira, 2023). Similarly, among formal caregivers in social welfare settings, greater job satisfaction correlates with improved quality of life and reduced absenteeism (Sousa et al., 2024). Additionally, in health and care professions in Portugal, social support and work engagement are significant predictors of job satisfaction, underscoring their vital role in maintaining workforce well-being (Orgambídez-Ramos & de Almeida, 2017).

Therefore, this study aims to evaluate the validity and factorial structure of the affective job satisfaction scale (BIASJ) in a sample of Portuguese IPSS workers. Although the scale was adapted to the Portuguese in 2015, this version was administered to workers from private institutions of social solidarity.

The main contribution of this study is in extending the validation of the BIAJS to a new and highly relevant professional group—IPSS workers in Portugal. While previous validations have been conducted with general working populations in other countries (Fernández-Muñoz & Topa, 2018; Uristemova et al., 2023), none focused on professionals in social solidarity institutions, who face intense emotional demands. By confirming the scale’s psychometric suitability in this context, this research provides empirical evidence of the BIAJS’s applicability in emotionally demanding organizational settings and supports its cross-cultural validity.

## 2. Method

### 2.1. Sample

The study sample included 234 IPSS workers; 92.3% were women. The participants had an average age of 39.15 years (SD = 8.22), with ages ranging from 22 to 66 years. Regarding marital status, most were married or in a domestic partnership (66.7%), and more than half had children (64.1%), mainly one or two. Most lived with others (90.6%). In terms of education, 47.4% held a high school diploma or an undergraduate degree. Lastly, 90% were permanent employees, with the majority working in nursing homes (66.2%) or day centers (53.8%).

The academic background of the sample mainly includes bachelor’s or licentiate degrees (n = 111, 47.4%), with the most common field of study being “social work” (27.4%), followed by “social education” (12.8%). When examining the contractual status of the participants, 90.2% are permanent employees, only 8% (n = 19) are on fixed-term or temporary contracts, and about 1.8% are self-employed. Regarding the social responses of the IPSS where the participants work, 66.2% report working in a residential structure for elderly people (ERPI); 53.8% work in a day center; 47% are part of the home support service; 11.1% are assigned to a social center; and 4.3% work in a night center. Concerning the number of training hours received by the participants in this research, about 51% report receiving less than 35 h of training, while roughly 25% attended more than 40 h.

### 2.2. Instruments

**Brief index of affective job satisfaction (BIAJS)** (Thompson & Phua, 2012) is a psychological tool designed to measure affective job satisfaction. It has a unidimensional structure composed of four items: “I find real enjoyment in my job,” “I like my job better than the average person,” “Most days, I am enthusiastic about my job,” and “I feel fairly well satisfied with my job.” The response scale is a 5-point Likert scale from 1 (strongly disagree) to 5 (strongly agree). The instrument includes three distractors to reduce acquiescence bias, such as: “My job needs me to be fit” and “My job is time-consuming.” The scale has been adapted and validated in various contexts, including among the Spanish population by Fernández-Muñoz and Topa (2018) with an internal consistency index of 0.847 (males) and 0.884 (females).

**The Maslach burnout inventory (MBI)** (Maslach et al., 1996) is a scale used to analyze burnout in professional environments. For this study, we only selected two factors from the scale: the first measures emotional exhaustion through five items with a total score of thirty points. This factor describes the feelings of a person emotionally exhausted by their work. The second factor measures depersonalization, which is assessed by five items, with a maximum total of thirty points. This factor describes an impersonal and cold response of professionals toward their clients. The response scale was a 7-point Likert scale, where participants rated how often they experience symptoms, ranging from 1 (never) to 7 (every day). The instrument has been validated in many organizational contexts and adapted in many countries, with internal consistency in all cases above 0.80.

### 2.3. Procedure

The research used a cross-sectional design, collecting data from 2023 to 2024 through a non-probabilistic sampling method. Participants were assured of their data’s anonymity and confidentiality in accordance with the General Data Protection Regulation. Participation (Assembleia da República, 2019) was voluntary. The inclusion criteria required participants to be over 18 years old, employed in private social solidarity institutions (PSSI) related to aging, have at least 1 year of professional experience in the aging sector, and live in Portugal. No specific professional fields were required. The questionnaire was distributed online via Google Forms, and the link was shared with a list of PSSI organizations participating in the study. These organizations were selected through incidental sampling. The study protocol was approved by the ethics committee of ANGES Associação Nacional de Gerontologia Social (Portugal-approved code: 20231).

### 2.4. Analysis

It is important to recognize that Hinkin’s (1998) five-step process is mainly designed for creating new measurement tools. In this study, the focus was not on developing a new scale but on validating the existing one (BIAJS) within a specific organizational and cultural setting. Therefore, although not all of Hinkin’s steps were suitable, the procedures used—exploratory and confirmatory factor analyses, reliability testing, and criterion validity assessment—align with standard practices for cross-cultural validation of established scales.

The statistical procedures were developed using JASP 0.18.3 (JASP Team, 2024). Assumptions including the KMO index, Bartlett’s test of sphericity (Kaiser, 1974), adequate sample size, multivariate normality, linearity, and variable correlation were verified for factor analysis (Tabachnick & Fidell, 1989). A varimax rotation method was employed for EFA, with factors identified through Horn’s parallel analysis (Horn, 1965); as the answer scale was a five-point ordinal, the approximation method was a robust estimator suitable for ordinal data: weighted least squares mean and variance adjusted (WLSMV). The internal consistency of the scale was assessed using Cronbach’s Alpha, McDonald’s omega, and item homogeneity.

We conducted a confirmatory factor analysis (CFA), with the same sample for the EFA, to assess model fit using various indices. Absolute fit indices included the chi-square statistic (X^2^), goodness-of-fit index (GFI) with scores over 0.90, and the standardized root mean square residual (SRMR), with values close to 0 being ideal. Incremental fit indices such as the comparative fit index (CFI), normed fit index (NFI), and incremental fit index (IFI) were also considered, with values above 0.90 indicating acceptability. Finally, the root mean square error of approximation (RMSEA) was evaluated, with values closer to 0 and a reference value of 0.08 indicating better fit (Bentler, 1990; Steiger & Lind, 1980).

## 3. Results

The internal consistency through Cronbach’s alpha of the BIASJ scale was α = 0.87 (CI 95% 0.84–0.90) and **McDonald’s omega** = 0.88. Table 1 shows the descriptive results (mean, standard deviation, skewness, and kurtosis), item homogeneity, Cronbach’s alpha and **McDonald’s omega**, and correlations between items. In this sense, item homogeneity was significantly elevated with a minimum value of 0.58 (item 2) and a maximum value of 0.80 (item 1 and item 3). Moreover, bivariate correlations between the items ranged from a minimum of *r* = 0.47 (between 2 and 4) to a maximum of *r* = 0.81 (between item 3 and item 4).

### 3.1. Exploratory and Confirmatory Factor Analysis

Regarding the validity of exploratory factor analysis (EFA), the Bartlett’s test of sphericity was *p* < 0.01 with a value of chi-square 555.26 (*df* = 6), and the sample index value of Kaiser–Meyer–Olkin (KMO) was 0.77. The factor weights from the exploratory factor solution were: “I find real enjoyment in my job” (item 1) = 0.86, “I like my job better than the average person” (item 2) = 0.62, “Most days, I am enthusiastic about my job” (item 3) = 0.88, and “I feel fairly well satisfied with my job” (item 4) = 0.84. The percentage of total variance explained for the one factor solution was 65%. The unidimensional model shows a marginal fit, due to the RMSEA, with scores over 0.08 (recommended value). Table 2 includes the indices values of the global scale: *X*^2^ = 5.43 *p* < 0.01 (*df* = 2), GFI = 0.99, AGFI = 0.98, CFI = 0.99, NFI = 0.99, IFI = 99, SRMR = 0.04, and RMSEA = 0.09.

### 3.2. Criterion Validity

The BIASJ scale correlated with the dimensions of Maslach’s scale (emotional exhaustion and depersonalization). The personal accomplishment as an inverse dimension was not included. Table 3 shows significant correlations with a confidence level of *p* < 0.01. Negative correlations were obtained between emotional exhaustion and BIASJ (*r* = −0.53) and depersonalization (*r* = −0.57). These correlations are supported by the previous literature where high levels of BIASJ are related to low levels of emotional exhaustions and depersonalization.

Although this study did not fully follow the five-step process outlined by Hinkin (1998), the core principles were incorporated. Specifically, the scale was administered to a relevant professional group, item homogeneity and internal consistency were tested, and both exploratory and confirmatory factor analyses were performed to assess factorial validity. Additionally, criterion validity was evaluated through correlations with theoretically related constructs. Therefore, even though not all Hinkin’s steps were implemented, the methodological rigor used provides strong evidence supporting the validity of the BIAJS in this context.

## 4. Discussion

The primary goal of this study was to examine the validity and factorial structure of BIASJ among workers at private institutions of social solidarity (IPSS) in Portugal. Affective job satisfaction is a crucial factor for psychological well-being within organizational settings and is linked to various positive outcomes for both employees and organizations (Warr, 2007; Fernández-Muñoz & Topa, 2018).

The results obtained provide evidence of the validity and reliability of BIASJ in a sample of IPSS workers in Portugal. The internal consistency of the scale, measured through Cronbach’s alpha and McDonald’s omega, was high, indicating that the items of the scale are coherent with each other and measure the same construct. These results were similar to those found by Uristemova et al. (2023) in a Kazakh version with academic faculty staff.

The factorial solution in this study supported the unidimensional structure of the scale, similar to Fernández-Muñoz and Topa (2018), although the RMSEA index did not reach the recommended values, indicating that there is still room to improve the model fit. The absolute and incremental fit indices, such as GFI, CFI, and NFI, showed acceptable values, supporting the adequacy of the proposed unidimensional model (Uristemova et al., 2023).

The criterion validity of the BIASJ scale was also demonstrated by significant negative correlations with the dimensions of emotional exhaustion and depersonalization on the Maslach scale. These findings align with previous research, indicating that higher affective job satisfaction is linked to lower levels of exhaustion and depersonalization (Topa & Alcover, 2015).

The findings of this study support the reliability and validity of the BIAJS in Portuguese IPSS contexts, confirming its unidimensional structure and negative correlations with burnout dimensions. These results are consistent with previous validations in different cultural contexts, such as Spain (Fernández-Muñoz & Topa, 2018), and Kazakhstan with university faculty (Uristemova et al., 2023). Compared to studies with younger populations (e.g., adolescents), the current findings emphasize the distinctiveness of emotional job satisfaction in professional, emotionally demanding environments, where the risk of burnout is particularly high. This indicates that the construct remains stable across different groups but may exhibit stronger associations with health-related outcomes in caregiving professions.

Despite the acceptable reliability scores, the poor RMSEA fit suggests that the unidimensional model may not fully represent the complexity of affective job satisfaction in high-demand organizational settings. This limitation highlights the need for future research to examine alternative models, potentially including second-order factors or cross-cultural adaptations.

This study has significant implications for both practice and policy. For organizations, especially IPSS, the BIAJS can be a simple yet effective tool to monitor workers’ emotional job satisfaction and detect early signs of burnout risk. Regular assessments could guide targeted interventions like training, supervision, and stress-reduction programs. For management, the results emphasize the importance of investing in organizational support and recognition systems that promote positive emotional experiences at work, which can help reduce turnover and absenteeism.

From a policy perspective, the findings highlight the importance of including well-being indicators in labor and mental health regulations for social care institutions. Given the rising demands on professionals working with vulnerable populations, instruments such as the BIAJS may help support evidence-based policymaking, ensuring better protection of workers’ psychological health.

This study has several limitations to consider when interpreting the results. First, the sample was collected through non-probability sampling, which can introduce biases in the psychometric analysis results; second, the sample has a significantly higher proportion of women than men, potentially affecting the generalization of the results to populations with a more balanced gender distribution; third, the cross-sectional design limits causal inferences; and finally, some indices, such as RMSEA, were higher than the recommended values, which could affect future validation results. Additionally, this study did not strictly adhere to Hinkin’s (1998) five-step process for scale development and validation. Instead, the focus was on the psychometric validation of an existing instrument, using procedures commonly employed in organizational psychology. While this simplifies Hinkin’s model, it should be noted that similar approaches have been used in other cross-cultural validations of the BIAJS (e.g., Fernández-Muñoz & Topa, 2018; Uristemova et al., 2023). Future research could strengthen the evidence by replicating these findings using the full five-step procedure.

In conclusion, the BIASJ scale can be considered, with certain limitations, a valid and reliable tool for assessing affective job satisfaction in IPSS contexts in Portugal. Nonetheless, future research should aim to include more diverse and gender-balanced samples and employ probability sampling methods to enhance the generalizability of the results.

## Figures and Tables

**Table 1:** Means, standard deviations, item homogeneity, if item is deleted, and inter-item correlation for the four items of the brief index of affective job satisfaction (BIAJS).

	M	SD	Skewness	Kurtosis	Item Homogeneity	1	2	3
BIAJS_1	4.10	0.95	−1.06	0.87	0.80			
BIAJS_2	3.50	1.07	−0.019	−0.069	0.58	0.63 *		
BIAJS_3	3.60	1.04	−0.049	−0.049	0.80	0.73 *	0.50 *	
BIAJS_4	3.54	1.08	−0.047	−0.047	0.76	0.69 *	0.47 *	0.81 *
Cronbach’s Alpha	0.87 (CI 95% 0.84–0.90)							
**McDonald’s Omega**	0.88 (CI 95% 0.85–0.90)							

* All correlations were significant at *p* < 0.01; answer scale from 1 to 5.

**Table 2:** Results of the Confirmatory Factor Analysis for the unidimensional solution of BIASJ.

	X^2^	df	*p*	GFI	AGFI	CFI	NFI	IFI	SRMR	RMSEA
Model F1	5.43	2	0.001	0.99	0.99	0.99	0.99	0.99	0.04	0.09

Note: GFI: goodness-of-fit index; AGFI: adjusted goodness-of-fit index; CFI: comparative fit index; NFI: normed fit index; IFI: incremental fit index; SRMR: standarized root mean square residual; and RMSEA: root mean square error of approximation.

**Table 3:** Correlations of the BIASJ with the dimensions of Maslach’s scale.

	1	2
BIASJ		
Emotional Exhaustion	−0.53 *	
Depersonalization	−0.57 *	0.71 *

* All correlations were significant at *p* < 0.01.

## Data Availability

The data supporting the findings of this study are available from ANGES, Associação Nacional de Gerontologia Social. Access to these data is restricted, as they were used under license for this study. Data may be obtained from the authors with the permission of ANGES, Associação Nacional de Gerontologia Social.

## References

[B1-ejihpe-15-00192] Assembleia da República (2019). Lei n.º 58/2019, de 8 de agosto: Assegura a execução, na ordem jurídica nacional, do Regulamento (UE) 2016/679 do Parlamento Europeu e do Conselho, relativo à proteção das pessoas singulares no que diz respeito ao tratamento de dados pessoais e à livre circulação desses dados.

[B2-ejihpe-15-00192] Bentler P. M. (1990). Comparative fit indexes in structural models. Psychological Bulletin.

[B3-ejihpe-15-00192] Brayfield A. H., Rothe H. F. (1951). An index of job satisfaction. Journal Applied Psychology.

[B4-ejihpe-15-00192] Fernández-Muñoz J. J., Topa G. (2018). Older workers and affective job satisfaction: Gender invariance in Spain. Frontiers in Psychology.

[B5-ejihpe-15-00192] Freudenberger H. J. (1974). Staff burnout. Journal of Social Issues.

[B6-ejihpe-15-00192] Hinkin T. R. (1998). A brief tutorial on the development of measures for use in survey questionnaires. Organizational Research Methods.

[B7-ejihpe-15-00192] Horn J. L. (1965). A rationale and test for the number of factors in factor analysis. Psychometrika.

[B8-ejihpe-15-00192] JASP Team (2024). JASP *(Version 0.18.3) [Computer software]. JASP Team*.

[B9-ejihpe-15-00192] Judge T. A., Kammeyer-Mueller J. D. (2012). Job attitudes. Annual Review of Psychology.

[B10-ejihpe-15-00192] Kaiser H. F. (1974). An index of factorial simplicity. Psychometrika.

[B11-ejihpe-15-00192] Lodi E., Zammitti A., Magnano P. (2021). Risk intelligence as a resource in career transition: The role of college satisfaction on the visions about future jobs. European Journal of Investigation in Health, Psychology and Education.

[B12-ejihpe-15-00192] Maslach C., Jackson S. E., Leiter M. P. (1996). Maslach burnout inventory manual.

[B13-ejihpe-15-00192] Maslach C., Leiter M. P. (2016). Understanding the burnout experience: Recent research and its implications for psychiatry. World Psychiatry.

[B14-ejihpe-15-00192] Maurício J., Laranjeira C. (2023). Job satisfaction and presenteeism: The mediating role of psychological distress in Portuguese private institutions of social solidarity. Administrative Sciences.

[B15-ejihpe-15-00192] Orgambídez-Ramos A., de Almeida H. (2017). Social support, work engagement and job satisfaction in Portuguese nursing staff: A cross-sectional study. Applied Nursing Research.

[B16-ejihpe-15-00192] Platania S., Caponnetto P., Morando M., Maglia M., Auditore R., Santisi G. (2021). Cross-cultural adaptation, psychometric properties and measurement invariance of the Italian version of the job satisfaction scale. European Journal of Investigation in Health, Psychology and Education.

[B17-ejihpe-15-00192] Platania S., Morando M., Gruttadauria S. V., Koopmans L. (2024). The individual work performance questionnaire: Psychometric properties of the Italian version. European Journal of Investigation in Health, Psychology and Education.

[B18-ejihpe-15-00192] Romano L., Consiglio P., Angelini G., Fiorilli C. (2021). Between academic resilience and burnout: The moderating role of satisfaction on school context relationships. European Journal of Investigation in Health, Psychology and Education.

[B19-ejihpe-15-00192] Sousa L., Santos A., Marques C., Correia C., Silva R. (2024). The impact of job satisfaction on the quality of life of formal caregivers of the elderly. Healthcare.

[B20-ejihpe-15-00192] Spector P. E. (1997). Job satisfaction: Application, assessment, causes, and consequences.

[B21-ejihpe-15-00192] Steiger J. H., Lind J. C. (1980). Statistically based tests for the number of common factors. Annual Spring Meeting of the Psychometric Society.

[B22-ejihpe-15-00192] Tabachnick B. G., Fidell L. S. (1989). Using multivariate statistics.

[B23-ejihpe-15-00192] Thompson E. R., Phua F. T. T. (2012). A brief index of affective job satisfaction. Group & Organization Management.

[B24-ejihpe-15-00192] Topa G., Alcover C. M. (2015). Psychosocial factors in retirement intentions and adjustment: A multi-sample study. Career Development International.

[B25-ejihpe-15-00192] Uristemova A., Myssayev A., Meirmanov S., Migina L., Pak L., Baibussinova A. (2023). Validation of the Kazakh version of the brief index of affective job satisfaction in medical universities faculty staff sample. Journal of Clinical Medicine of Kazakhstan.

[B26-ejihpe-15-00192] Warr P. (2007). Work, happiness, and unhappiness.

